# Dynamics and consequences of nutrition-related microbial dysbiosis in early life: study protocol of the VITERBI GUT project

**DOI:** 10.3389/fnut.2023.1111478

**Published:** 2023-05-18

**Authors:** Jeanne Tamarelle, Margaux M. Creze, Vanthanom Savathdy, Sengrloun Phonekeo, Jordyn Wallenborn, Latsamy Siengsounthone, Günther Fink, Peter Odermatt, Sengchanh Kounnavong, Somphou Sayasone, Pascale Vonaesch

**Affiliations:** ^1^Department of Fundamental Microbiology, University of Lausanne, Lausanne, Switzerland; ^2^Lao Tropical and Public Health Institute, Ministry of Health, Vientiane, Lao People’s Democratic Republic (PDR); ^3^Department of Epidemiology and Public Health, Swiss Tropical and Public Health Institute, Allschwil, Switzerland; ^4^University of Basel, Basel, Switzerland

**Keywords:** overnutrition, undernutrition, metabolic syndrome, epigenetics, microbiota inheritance, microbial succession, Lao PDR, maternal and child health

## Abstract

**Introduction:**

Early life under- and overnutrition (jointly termed malnutrition) is increasingly recognized as an important risk factor for adult obesity and metabolic syndrome, a diet-related cluster of conditions including high blood sugar, fat and cholesterol. Nevertheless, the exact factors linking early life malnutrition with metabolic syndrome remain poorly characterized. We hypothesize that the microbiota plays a crucial role in this trajectory and that the pathophysiological mechanisms underlying under- and overnutrition are, to some extent, shared. We further hypothesize that a “dysbiotic seed microbiota” is transmitted to children during the birth process, altering the children’s microbiota composition and metabolic health. The overall objective of this project is to understand the precise causes and biological mechanisms linking prenatal or early life under- or overnutrition with the predisposition to develop overnutrition and/or metabolic disease in later life, as well as to investigate the possibility of a dysbiotic seed microbiota inheritance in the context of maternal malnutrition.

**Methods/design:**

VITERBI GUT is a prospective birth cohort allowing to study the link between early life malnutrition, the microbiota and metabolic health. VITERBI GUT will include 100 undernourished, 100 normally nourished and 100 overnourished pregnant women living in Vientiane, Lao People’s Democratic Republic (PDR). Women will be recruited during their third trimester of pregnancy and followed with their child until its second birthday. Anthropometric, clinical, metabolic and nutritional data are collected from both the mother and the child. The microbiota composition of maternal and child’s fecal and oral samples as well as maternal vaginal and breast milk samples will be determined using amplicon and shotgun metagenomic sequencing. Epigenetic modifications and lipid profiles will be assessed in the child’s blood at 2 years of age. We will investigate for possible associations between metabolic health, epigenetics, and microbial changes.

**Discussion:**

We expect the VITERBI GUT project to contribute to the emerging literature linking the early life microbiota, epigenetic changes and growth/metabolic health. We also expect this project to give new (molecular) insights into the mechanisms linking malnutrition-induced early life dysbiosis and metabolic health in later life, opening new avenues for microbiota-engineering using microbiota-targeted interventions.

## 1. Introduction

### 1.1. Epidemiology of undernutrition, overnutrition and diet-related non-communicable diseases

Metabolic syndrome (MetS) is a diet-related cluster of conditions that frequently co-occur and increase a person’s risk of heart disease, stroke, and type 2 diabetes (T2D), which are among the leading causes of death worldwide (1). These conditions include increased blood pressure, high blood sugar, excess visceral body fat and abnormal cholesterol or triglyceride levels in the blood (2). It is now well-established that overweight and obesity can lead to MetS (3), which is problematic given the prevalence of obesity and overweight worldwide. According to the World Health Organization, in 2016, approximately 1.9 billion adults were overweight or obese (4). Numbers are also rapidly rising among children under the age of five with 39 million children considered overweight and obese in 2020 (5).

There is increasing evidence that caloric restriction and undernutrition is also associated with the development of diet-related non-communicable diseases (DR-NCD) such as MetS or T2D (6–8), especially when undernutrition occurs in the first years of life and is followed by a rapid weight gain after infancy (9). Latest estimates suggest that there are 144 million stunted children, 47 million wasted children and 340 million micronutrient-deficient children worldwide (10); and these numbers are unlikely to improve in the short run given the COVID-19-related increase in poverty and food insecurities across the world (11). It is hypothesized that the unexpected association between undernutrition and DR-NCD is due to the exposure to nutrient-dense food following former undernutrition, thus imposing a high metabolic load on a given subject (12). The co-occurrence of undernutrition and overnutrition within the same countries, families and even individuals is termed double burden of malnutrition.

### 1.2. Situation in Asia/Laos

In Asia, MetS prevalence ranges from 10 to 50%, similar to other parts of the world, and has steadily increased since the nineties (13). Further, in Asia, T2D has developed over a much shorter time, in a younger age group, and in people with much lower body mass indexes compared to other parts of the world (14). Indeed, Asian countries have experienced a recent increase in economic growth with easier access to and over-compensation with nutrient-rich food, which could lead to an explosion of metabolic diseases in the decades to come. In 2019, Asia was home to about half of the world’s overweight or obese children under 5 years of age (5). Further, in Laos, 33% of all children under 5 year of age are stunted, many of them even before weaning [*Vientiane: Lao Statistics Bureau and UNICEF* (15)].

### 1.3. Underlying mechanisms of the double burden of malnutrition and the thrifty phenotype hypothesis

A meta-analysis of 14 studies including 132,180 subjects found a U-shaped pattern between birth weight and the occurrence of T2D in adulthood: both birth weight below 2,500 g and over 4,000 g were associated with high risk of T2D compared with normal birth weight (16). One of the proposed mechanisms is that catch-up growth is characterized by hyperinsulinemia and a higher rate of body fat recovery compared to lean tissue (preferential “catch-up fat”). Energy conservation (thrifty) mechanisms can direct glucose preferentially toward *de novo* lipogenesis and storage in white adipose tissue (“thrifty phenotype hypothesis”). The body can further adapt to the changed nutrient availability through epigenetic mechanisms, which include histone modifications or DNA methylation, and lead to alterations in gene expression. Epigenetic developmental plasticity is especially pronounced during fetal life, therefore increasing the adaptability of the newborn to its environment (17). In recent years, mostly through work using animal models, a clear link between prenatal and early life under- and overnutrition and epigenetic changes was described (18–20). In addition, an association between early life epigenetic changes and later metabolic risk was unraveled (21).

### 1.4. Prenatal and early life developmental window

The association between birth weight and chronic diseases, such as obesity, T2D, cardiovascular disease, stroke and hypertension, was originally proposed by David Barker, who first formulated the hypothesis that insults in fetal or early life are linked to adult chronic diseases (22–24). This hypothesis was later extended to the developmental origin of health and disease (DOHaD) theory (25), which states that the period from conception to the second birthday (“first 1,000 days”) is the most important developmental window to ensure healthy growth and prevent disease later in life [reviewed in Popkin et al. (26)]. These first 1,000 days also correspond to the critical window for nutritional interventions (27) as they are very important for organ development and are a crucial time when the microbiota establishes and experiences an ecological succession (28). The intestinal microbiota comprises pro- and eukaryotic microorganisms as well as viruses and phages, which altogether encode 100 times more genes than we harbor in our own genome (29). The intestinal microbiota is acquired at birth through exposure to the vaginal and fecal microbiome of the mother and gradually changes in the first years of life until it reaches stability when the child is about 3 years old [reviewed in (30)]. It has been postulated that the maternal microbial intrauterine environment and early microbial colonization of the child could play an important role as mediators in the DOHaD (31) hypothesis. However, to date, there is only little data to support this hypothesis (32).

### 1.5. Fecal microbiota changes and implication in metabolic health and diet-related non-communicable diseases

A growing body of evidence has highlighted the central role of the intestinal microbiota in understanding physiological responses to nutritional intake. Fecal microbiota transplant experiments in animals have shown that the fecal microbiota is causally implicated in weight gain (33) as well as in undernutrition (34, 35). Further, it has been demonstrated that microbiota transfer from lean human donors into patients suffering from MetS improves insulin sensitivity (36), suggesting that the microbiota plays a causal role in MetS. Overall, in obese patients, a decreased bacterial diversity with lower relative abundance of several bacterial groups such as *Clostridiales*, *Akkermansia*, *Bifidobacterium*, and *Methanobrevibacter*, and a higher relative abundance of *Streptococcus, Blautia, Bilophila wadsworthia*, and *Enterobacteriaceae* have been observed [reviewed in Tilg et al. (37)]. Interestingly, similar effects, including a decrease in microbial diversity and butyrate production, have been observed in subjects displaying a pre-metabolic state (38) and/or MetS (39).

**FIGURE 1:**
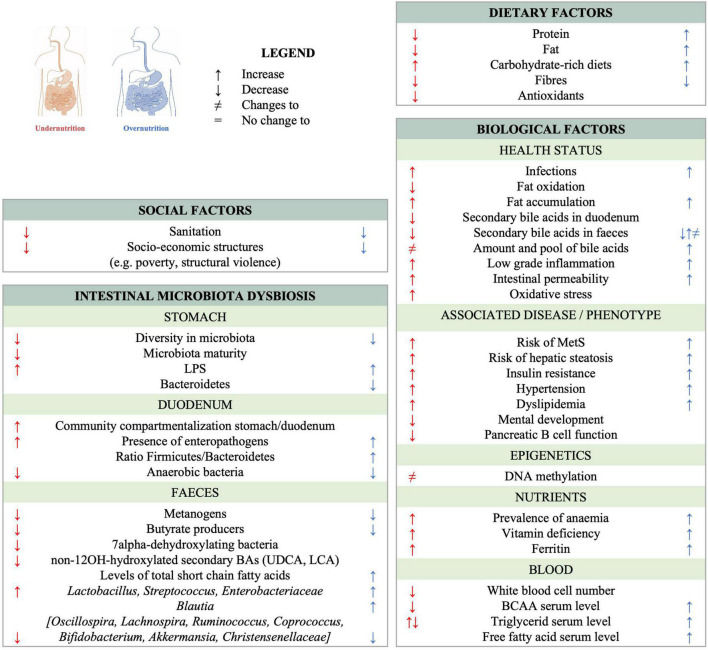
Observed changes to the gut ecosystem and shared characteristics between chronic undernutrition and overnutrition/obesity. References can be found in Supplementary Table 1.

Undernutrition and obesity share several microbiota-associated characteristics (Figure 1). Changes in the pool of bile acids occur in both under- and overnutrition (40, 41) and both conditions also lead to significant changes in the fecal microbiota. Some of these changes are specific to a given form of malnutrition, while others, such as the decrease of butyrate-producing *Clostridia*, are observed in both undernutrition and MetS (37, 42, 43). Of note, supplementation with butyrate has been shown to be beneficial in the context of MetS as reviewed in van Deuren et al. (44), Bridgeman et al. (45) and Coppola et al. (46). Further, *Bilophila wadsworthia* is overrepresented in both acutely undernourished subjects (35) and subjects suffering from MetS (47). *B. wadsworthia* induces inflammation, contributes to gut permeability, and aggravates the metabolic phenotype in MetS patients. Moreover, ectopic colonization by *Streptococci* and/or other taxa normally residing in the oro-pharyngeal tract has been observed in the context of T2D (48), liver disease (49), inflammation-related chronic diseases (50–52), as well as chronic undernutrition (42). Under- and overnutrition also share additional patterns which are not related to the microbiota, of which several are related with a higher risk for metabolic syndrome: both are associated with carbohydrate-rich diets, micronutrient deficiencies, increased oxidative stress and altered bile acid profiles as reviewed in (53). Therefore, it seems plausible that the etiological pathway from early life under- and overnutrition to MetS is at least in part shared and can thus be addressed with joint intervention and prevention strategies.

### 1.6. Objectives

#### 1.6.1. Overall objective and hypotheses of this study

The overarching goal of the VITERBI GUT project is to identify the causes and biological mechanisms that link prenatal or early life under- or overnutrition with pre-disposition to develop obesity and metabolic disease in later life. To this purpose, we will evaluate the contribution of the maternal and child’s nutritional status (both under- and overnutrition) as well as their microbiome composition during pregnancy/fetal life and the first months of life, encompassing the first 1,000 days of life. We hypothesize that the double burden of malnutrition is due to the inheritance of a dysbiotic microbiota at birth, making the children vulnerable to obesity and MetS in later life. We further hypothesize that under- or overnourished pregnant mothers display a dysbiotic oral, vaginal, and/or fecal microbiota, which is the source of a “dysbiotic vertical transmission.” Further, we hypothesize that similar taxa and functions may be affected in children from both chronically undernourished and obese mothers and that the link between early life exposures and the child’s metabolic development are mediated at least in part by epigenetic changes. Finally, we postulate that the trajectory of the child’s microbiota is influenced by breastfeeding initiation, exclusivity and length, which may, at least partially, overcome the dysbiotic seeding at birth.

#### 1.6.2. Specific objectives/research questions

**FIGURE 2:**
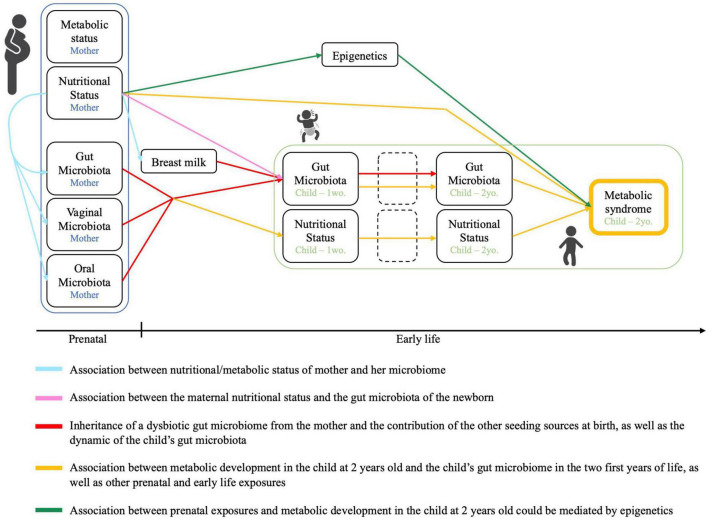
Conceptual framework and objectives of the VITERBI GUT prospective birth cohort. Created using BioRender.com.

VITERBI GUT has the following objectives (Figure 2): First, we will investigate the association between nutritional/metabolic status and the oral, vaginal, fecal, and breast milk microbiome in (pregnant) mothers. Second, we will investigate for an inheritance of a dysbiotic microbiota at birth by assessing for the association between the maternal nutritional status and the fecal (seed) microbiota of the newborn infant. We will also evaluate the contribution of other seeding sources including the maternal oral and vaginal microbiota as well as breast milk microbiota to the infant’s microbiota. Third, we will assess for bacterial succession of the child’s fecal microbiota over the first 2 years of life in the context of maternal over- and undernutrition and assess for a potential modulating effect of breastfeeding. Fourth, we will evaluate the association between the metabolic profile of the child at 2 years of age and the child’s early life microbiota succession, as well as other prenatal and early life exposures. Last, we will investigate for a potential association between prenatal exposures, the metabolic health of the child at 2 years of age and changes to the blood DNA methylome. Metabolic health of the child will be based on the definition of MetS provided by the International Diabetes Federation. For children above 10 years of age, MetS is defined as a combination of obesity (defined through waist circumference) and at least 2 of the following 4 criteria: high blood triglyceride levels, low HDL-Cholesterol levels, high blood pressure and high fasting glucose based on age-specific thresholds. In children below the age of 10 years, there is no official definition for MetS. To get an estimation of the child’s metabolic health, we will evaluate each of these five criteria individually and in combination when the children are 2 years old. Further, we will assess for birth weight, skinfold thickness and BMI as additional secondary and intermediate outcomes.

Taken together, these objectives will allow us to delineate the microbiota trajectory in mother-child dyads in detail and to assess the early life factors shaping metabolic health in early childhood.

## 2. Materials and methods

### 2.1. Study design/setting

**FIGURE 3:**
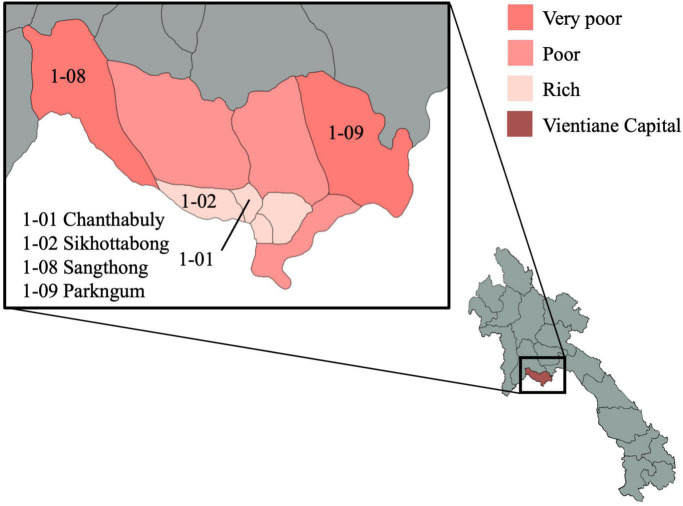
Map of the study districts in Vientiane capital province. The selected districts for VITERBI GUT are Chanthabuly (1-01), Sikhottabong (1-02), Sangthong (1-08), and Parkngum (1-09). The maps were generated with GADM data (gadm.org, version 4.0.4) and the magrit application (magrit.cnrs.fr, version 0.8.14).

The VITERBI GUT project is a longitudinal prospective birth cohort of children born to undernourished, overnourished and normally nourished mothers. The project is nested in the VIentiane mulTi gEneRational BIrth Cohort (VITERBI), jointly led by the Lao Tropical and Public Health Institute (Lao TPHI) and the Swiss Tropical and Public Health Institute (Swiss TPH). VITERBI aims at quantifying key health challenges and gaps in Vientiane Capital to later develop interventions and programs to improve population health. VITERBI is designed to be representative of the population of Vientiane, Lao People’s Democratic Republic (PDR), and enrolls participants in four districts, which are selected based on their socioeconomic status and the urban/rural setting: Chanthabuly and Sikhottabong as two urban districts with high socioeconomic status, and Sangthong and Parkngum as rural districts with low socio-economic status (Figure 3).

### 2.2. Participants

In total, 300 mother-child dyads will be recruited from the pool of VITERBI participants, of which 100 mothers are undernourished, 100 are overnourished and 100 are normally nourished. Pregnant mothers will be classified as under-, over- or normally nourished based on their mid-upper arm circumference (MUAC). According to the cut-off suggested by Ververs (54), undernourished women will be defined as having a MUAC < 23 cm, and overnourished women will be defined as having a MUAC > 27 cm. Women with a MUAC between 23 and 27 cm will be considered as normally nourished. Mothers eligible for VITERBI GUT have to be previously included in the multigenerational cohort VITERBI, thus living in Chanthabuly, Sikhottabong, Sangthong, or Parkngum and should not plan to move out of these four districts. Further, they should be older than 18 years of age and provide written consent to participate in the study. Mothers that are self-declared HIV positive will be excluded from the study.

### 2.3. Recruitment, follow-up, and data collection

#### 2.3.1. Recruitment procedures

Recruitment started in March 2022 and is expected to last for 1 year. Women are recruited during the third trimester of pregnancy (inclusion visit) and are followed-up together with their child at five timespoints until the child reaches the age of 2 years. Completion of the study is planned for May 2025.

Pregnant women from the pool of VITERBI participants are screened based on their age, expected date of delivery, and MUAC values to establish a list of eligible women. Pregnant women who potentially fit the inclusion criteria are contacted and a pre-inclusion visit is scheduled for the last trimester of their pregnancy, ideally around the 8st month of pregnancy. During the pre-inclusion visit, the mothers are informed about the study, the inclusion criteria are checked, and, if eligible, the pregnant women are invited to participate to the study and sign an informed consent form.

#### 2.3.2. Inclusion and follow-up visits

**FIGURE 4:**
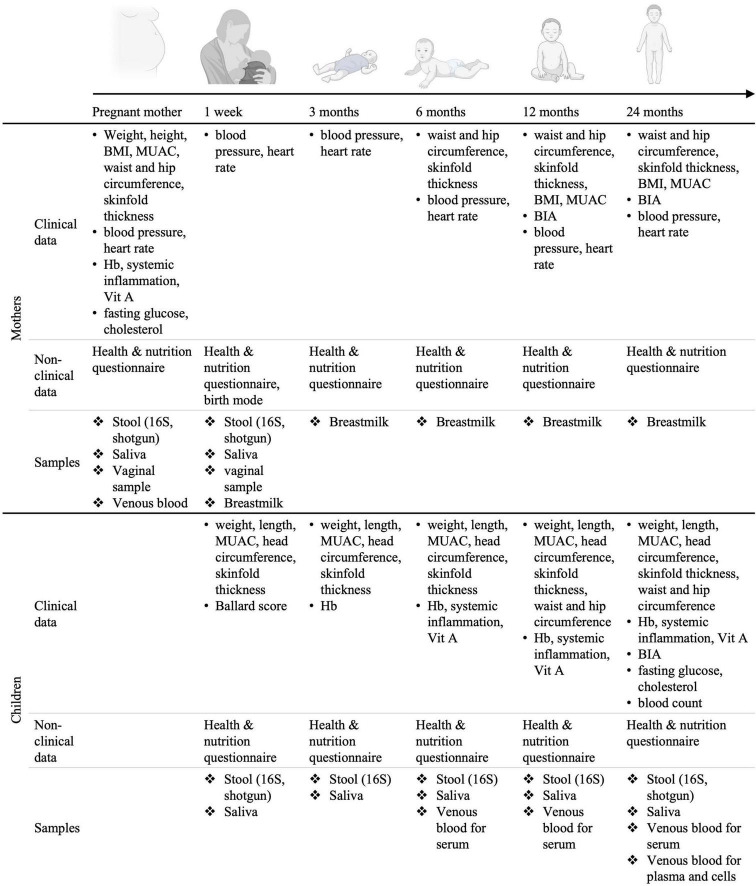
Set-up of the longitudinal birth cohort study in Lao PDR. Mother-child dyads will be followed for 2 years post-partum. BMI, body mass index; MUAC, middle-upper arm circumference; Hb, hemoglobin measurement; Vit. A, Vitamin A; BIA, bioimpedance analysis. Created using BioRender.com.

The inclusion visit takes place in the third trimester of pregnancy, shortly after the pre-inclusion visit. A clinical team composed of a nurse and a lab technician visits the pregnant women at home or in the village office, fills out a digital questionnaire and collects all needed biological specimens. An appointment is made for the 1-week post-partum visit based on the expected date of delivery. Follow-up visits are planned at 1-week, 3, 6, 12, and 24 months post-partum. At each follow-up visit clinical, nutritional and anthropometric information about the mothers and the children is collected (Figure 4). The dates of the follow-up visits are re-adjusted once the child is born.

#### 2.3.3. Collection of primary (clinical, nutritional, and household data) and secondary data (analyses of biospecimens)

During each visit, a clinical assessment of the mother’s and child’s general health status is performed. Clinical data such as anthropometric measurements, body composition and anemia are collected at different time points during the follow-up visits as specified in Figure 4. Non-clinical data regarding nutrition and household characteristics is collected through the administration of custom-made questionnaires. The questionnaires are separated in six parts: “General information,” “Mother’s health,” “Child’s health,” “Mother’s nutrition,” “Breastfeeding and child’s nutrition,” and “Water and sanitation.” The questionnaire regarding nutrition is based on the 24 h recall, the other questionnaires were custom made and aim at reflecting the most likely factors influencing microbiota composition and maternal and child health outcomes. Data is entered in an open-source app [Open Data Kit (55)]. In addition, to assess the mother’s and child’s metabolic health and microbiome, biospecimens are collected at each time point for further analyses, generating secondary data such as blood lipid profiles, vitamin levels, inflammation biomarkers, information on infestation by parasites, microbiome composition and blood methylome profile (Figure 4). All the primary and secondary data generated is stored on encrypted hardwares or on fileservers with customizable access rights.

### 2.4. Materials/laboratory methods

#### 2.4.1. Biospecimen collection

Fresh stool samples for parasitic analysis are kept at 4°C. One aliquot is used for immediate analysis using the Kato-Katz and direct smear techniques and another one is stored in a Sodium Acetate-Acetic Acid-Formaline solution (back-up). Additional stool samples are stored for DNA extraction, metabolite analysis and bacterial isolation (glycerol stocks). Saliva and vaginal samples are self-collected using Salimetrics saliva collection swabs (Salimetrics, Carlsbad, USA) and COPAN eSwab collection kits (COPAN Diagnostics, Murrieta, CA, USA), respectively. Vaginal samples are collected using a dry swab and stored in 1 ml of DNA/RNA shield stabilization solution (Zymoresearch, Irvine, CA, USA). Breast milk is hand expressed by the mother directly into a sterile collection tube without additive. The milk is collected from a single breast and the latter is entirely emptied to collect the full feed. The milk is thoroughly mixed and an aliquot of up to 50 ml is stored for later analysis. The collection is performed at least 2 h after the mother reports to have breastfed her baby for the last time. Once collected, the milk is immediately stored in a portable −20°C freezer and transferred to −80°C within 6 h. The sample collection is accompanied with a tracking sheet recording the time the sample spent at −20°C as well as the overall volume of milk collected. Blood collection is performed by a trained nurse using a Vacutainer (BD Vacutainer^®^ Safety-Lok™) both for mothers and children. Serum samples are obtained by centrifugation (directly in the field) and used to measure metabolic, nutritional and inflammation biomarkers. An additional blood sample will be collected from the children at 2 years of age using EDTA tubes and will be used for a general blood count and epigenetic analyses. Blood samples will be stored immediately at 4°C (whole blood) or −20°C (serum) in a portable freezer/fridge and transferred to the laboratory for further processing or storage at −80°C within 6 h. All samples are accompanied with a tracking sheet recording the time the sample spent at 4°C/−20°C as well as the number of tubes/overall volume collected.

#### 2.4.2. DNA extraction and sequencing methods

DNA extraction will be performed on stool, saliva, breast milk and vaginal samples according to previously established and standardized protocols. Characterization of the microbiota will be performed using amplicon sequencing of the hypervariable region of the 16S rRNA gene. Shotgun metagenomic sequencing will be performed on a subset of fecal samples to allow strain inference as well as determination of the metabolic potential of a given microbial community.

#### 2.4.3. Characterization of the microbiome

Bioinformatic analyses will be performed according to standard procedures in the field and adapted to new tools widely used at the time of data analysis, i.e., using the Dada2 package (56) and the Silva Reference Database (57) for amplicon sequences, the KneadData Tool for pre-cleaning of the shotgun metagenomics data, HUMAnN (58) for inferring gene and pathway abundance and MetaPhlAn (59, 60) and PhyloPhlAn (61) for abundance of specific bacterial species and strains.

#### 2.4.4. Analysis of the DNA methylome

The blood whole genome DNA methylome of the child at 2 years of age will be characterized using an Illumina MethylationEPIC BeadChip in peripheral blood mononuclear cells (PBMCs). PBMCs are an easily accessible surrogate tissue and, as many metabolites circulate through the blood, good “sensors” for metabolites reaching systemic sites. The analysis will be performed as outlined in Jeong et al. (62) and Imboden et al. (63). Briefly, DNA/RNA will be isolated, quality controlled, bisulfite converted, amplified, fragmented and hybridized to an Illumina MethylationEPIC BeadChip. The obtained data will then be quality controlled and annotated. Data will be normalized using the R package R minfi (64) and background corrected using Noob (normal-exponential out-of-band) (65). PBMCs are a heterogeneous mixture of epigenetically distinct cell types, with varying cell counts between individuals. We will therefore adjust the results for the basic cell-count using multivariate regression and specialized statistical modeling approaches (66–69).

### 2.5. Statistical considerations

#### 2.5.1. Statistical methods

Statistical analyses will be performed in the R statistical environment. Associations between microbiome features (taxa or genes) and clinical outcomes (such as the nutritional status of the mother, her metabolic status, the nutritional and metabolic status of the child) will be explored using measures of microbial diversity (α-diversity and β-diversity scores), community analyses (i.e., through ecotypes or guilds) and differential abundance testing of specific features and bacteria. Compositional differences in terms of bacterial taxa will be visualized using principal coordinates analysis (PCoA) and tested with multivariate PERMANOVA analysis as well as different modeling approaches. Differences in the bacterial composition and abundance between children born to under-, normally or over-nourished mothers will be evaluated using differential abundance testing. Microbial signatures will be confirmed by performing targeted qPCR for the respective bacterial taxa. All models will account for confounding factors, such as sequencing batch and depth or host variables (sex, age, BMI, food intake, …) (70).

The transmission of given bacterial species from the mothers to their children will be assessed by comparing the mother’s fecal microbiota shortly before birth to the child’s fecal microbiota at 1 week of age. Bacterial strains associated with or shared between the child’s fecal microbiota and the mother’s microbiota from different body sites (stool, saliva, vaginal sample, and breast milk) will be evaluated at each time point and compared to unrelated mother-child dyads, as previously described (71–73).

For DNA methylation analyses, the association of single CpG markers with the child’s metabolic status as well as other factors such as maternal nutritional and metabolic status (blood glucose, lipid profile, and blood pressure) and mode of delivery will be assessed through epigenome-wide covariate-adjusted linear regression and region-based analysis. We will further statistically account for correlations between adjacent probes. The correlation between given CpG markers and microbial taxa in the fecal or vaginal microbiota of the mothers will be analyzed through canonical correlation analyses, network-based statistical methods and other advanced modeling approaches.

#### 2.5.2. Sample size estimation

For sample size calculation, we used the expected proportions of pre-MetS found in children born to undernourished, normally nourished and overnourished women at the age of 2 years. We define pre-MetS as a combination of obesity (defined through waist circumference) and at least two of the following four criteria: high blood triglyceride levels, low HDL-Cholesterol levels, high blood pressure and high fasting glucose based on age-specific thresholds. Prevalences for pre-MetS are not published/available. We thus estimated that children born to normally-nourished women would have a similar proportion of pre-MetS to non-obese/non-overweight children, i.e., approximately 1%, based on a systematic review of the literature by Friend et al. (74). For children born to undernourished and overnourished mothers, we hypothesized that the proportion developing pre-MetS would be lower than what is found in the literature for children that are overweight or obese themselves, i.e., about 12% for overweight and 29% for obese children. Specifically, we anticipate a pre-MetS prevalence of 10% for children born to under- or overnourished mothers. With an alpha of 5%, 100 mother/child pairs are needed to reach power 0.8. In a previous study by Vonaesch et al. (42), the microbiota of 400 children, stratified into subgroups consisting of at least 36 samples was assessed. The group was able to demonstrate statistical significant changes in given taxa, which are conserved in the two study sites in all subgroups even when adjusting for multiple testing and correcting for different confounding factors. Based on these data, a sample size of 300 mother-infant dyads should be sufficient to assess microbiota changes in the three different groups.

## 3. Discussion

Under- and overnutrition have both been associated with MetS and T2D later in life. As there is still a large proportion of women across the world suffering from these syndromes, it is of utmost scientific and public health importance to better understand the biological factors that affect the risk of developing MetS and T2D. Further, despite the well-known fact that the microbiota composition differs on a global scale (75–80) most data to date comes from high income countries and we lack data on low-and-middle income countries (LMIC), including in African and South-East Asia (80–83). In December 2019, The Lancet launched a special series dedicated to the double burden of malnutrition, highlighting the growing interest in this subject (26, 84, 85). Historically, interventions addressing undernutrition and obesity have been developed and delivered separately from one another. Recognizing the interconnections between these two forms of malnutrition might reveal new, shared opportunities to improve metabolic health. This is of major importance as evidence shows that programs addressing undernutrition have unintentionally increased risks for obesity and diet-related non-communicable diseases (DR-NCDs) in LMICs (86) where food environments are changing rapidly (85).

VITERBI GUT will be the first study to look at early life microbiota and metabolic health in Lao PDR, a country still largely affected by undernutrition and where increasing numbers of mothers are suffering from overweight and obesity. Overall, VITERBI GUT will establish a comprehensive framework of prenatal and early life determinants of metabolic health by investigating the relative contribution of the mother’s nutritional and metabolic status as well as the maternal and child’s microbiota.

In children and adults, the microbiota is to some extent correlated across body sites (87), in particular in the context of undernutrition, where previous studies have shown an homogenization of the bacterial communities between the oral cavity and the intestinal environment (42, 88). At birth, the microbiome of children is immature and will progressively adapt to its niche to reach a mature state at around 2 years of age. However, in malnourished children, the microbiome remains immature for prolonged periods of time (89). It has been shown that the maternal microbiota is associated with postnatal growth (90). Further, previous studies have established strain sharing between the healthy mothers and their children (71). VITERBI GUT expands on this work by taking into consideration the maternal nutritional and metabolic status prior to birth and by following children until the age of 2 years to assess the long-term influence on the child’s metabolic and epigenetic profile. As DR-NCDs show an intergenerational pattern, successful interventions might need to begin before conception, most likely in all women of childbearing age, and continue throughout the pregnancy and lactation period. By delineating the direct and indirect effects of the mother’s nutritional status, the mother’s microbiome, the child’s early life microbiome, as well as DNA methylation and other important determinants, the study represents an important step toward developing efficient, evidence-based intervention strategies.

VITERBI GUT builds on an existing multi-generational cohort including pregnant women in four districts of the Vientiane Prefecture (VITERBI project). The districts were chosen based on their socioeconomic level, the two poorest and the two richest districts of the Vientiane Prefecture, while staying fairly accessible for logistic reasons. The representativeness and hence the generalizability of our results might be affected by this choice. To validate part of our results on a different population, we will compare the results from the Laotian cohort with the GENEIDA (Genetics, Early Life Environmental Exposures and Child Development in Andalucìa) prospective birth cohort, launched in 2014 in Almeria, Spain and led by Prof. Marina Lacasaña of the Andalusian School of Public Health. The GENEIDA cohort follows pregnant women and their children for up to 4 years post-partum, with oral, vaginal and fecal samples collected during pregnancy, and when the child is 12 and 24 months old. Clinical, anthropometric and nutritional data were collected for the mothers and the children. We will assess for the effect of maternal metabolic status and the maternal fecal and vaginal microbiota on the child’s metabolic health at 2 years of age in 100 normally nourished and 100 overnourished women. We will assess for shared associations to identify universal pathophysiological mechanisms underlying the trajectory from prenatal/early-life overnutrition to metabolic disease, which are independent of geography as well as the genetic or social background of the subjects.

Overall, the VITERBI GUT project will contribute important new insights into the role of prenatal/early life exposures in shaping the epigenetic and metabolic development of children, which are known to be crucial factors to ensure lifelong health.

## Ethics statement

This study was reviewed and approved by Ethics Committee of Northwestern and Central Switzerland (EKNZ, Switzerland) (AO_2021-00032), as well as the Lao PDR National Ethics Committee for Health Research (NECHR, Ministry of Health, Lao PDR). Written informed consent to participate in this study was provided by the participants legal guardian/next of kin.

## Author contributions

PV, SK, GF, JW, SS, and PO conceived the protocol. PV wrote the protocol. PV and JT acquired funding for the study. PV, JT, and MMC assured the overall steering and management of the study. SS, VS, and SP assured the coordination of the fieldwork. PV, SK, LS, and SS coordinated the study. JT, MMC, and PV wrote the present manuscript. All authors read and approved the final version of the manuscript.

## References

[1] Roth G Abate D Abate K Abay S Abbafati C Abbasi N Global, regional, and national age-sex-specific mortality for 282 causes of death in 195 countries and territories, 1980–2017: a systematic analysis for the Global Burden of Disease Study 2017. *Lancet.* (2018) 392:1736–88. 30496103 10.1016/S0140-6736(18)32203-7 PMC6227606

[2] Cani P Amar J Iglesias M Poggi M Knauf C Bastelica D Metabolic endotoxemia initiates obesity and insulin resistance. *Diabetes.* (2007) 56:1761–72. 10.2337/db06-1491 17456850

[3] Falkner B Cossrow N. Prevalence of Metabolic Syndrome and Obesity-Associated Hypertension in the Racial Ethnic Minorities of the United States. *Curr Hypertens Rep.* (2014) 16:449. 10.1007/s11906-014-0449-5 24819559 PMC4083846

[4] WHO. *Obesity.* Geneva: WHO (2022).

[5] WHO. *Obesity and overweight.* Geneva: WHO (2022).

[6] Victora C Adair L Fall C Hallal P Martorell R Richter L Maternal and child undernutrition: consequences for adult health and human capital. *Lancet.* (2008) 371:340–57. 10.1016/S0140-6736(07)61692-4 18206223 PMC2258311

[7] Eriksson J Kajantie E Lampl M Osmond C. Trajectories of body mass index amongst children who develop type 2 diabetes as adults. *J Intern Med.* (2015) 278:219–26. 10.1111/joim.12354 25683182

[8] Bhargava S Sachdev H Fall C Osmond C Lakshmy R Barker D Relation of serial changes in childhood body-mass index to impaired glucose tolerance in young adulthood. *N Engl J Med.* (2004) 350:865–75. 10.1056/NEJMoa035698 14985484 PMC3408694

[9] Arisaka O Ichikawa G Koyama S Sairenchi T. Childhood obesity: rapid weight gain in early childhood and subsequent cardiometabolic risk. *Clin Pediatr Endocrinol Case Rep Clin Investig.* (2020) 29:135–42. 10.1297/cpe.29.135 33088012 PMC7534524

[10] FAO. *The State of Food Security and Nutrition in the World 2020.* Rome: FAO (2022).

[11] Littlejohn P Finlay B. When a pandemic and an epidemic collide: COVID-19, gut microbiota, and the double burden of malnutrition. *BMC Med.* (2021) 19:31. 10.1186/s12916-021-01910-z 33504332 PMC7840385

[12] Dulloo A Jacquet J Seydoux J Montani J. The thrifty “catch-up fat” phenotype: its impact on insulin sensitivity during growth trajectories to obesity and metabolic syndrome. *Int J Obes.* (2006) 30(Suppl 4):S23–35. 10.1038/sj.ijo.0803516 17133232

[13] Ranasinghe P Mathangasinghe Y Jayawardena R Hills A Misra A. Prevalence and trends of metabolic syndrome among adults in the asia-pacific region: a systematic review. *BMC Public Health.* (2017) 17:101. 10.1186/s12889-017-4041-1 28109251 PMC5251315

[14] Yoon K Lee J Kim J Cho J Choi Y Ko S Epidemic obesity and type 2 diabetes in Asia. *Lancet Lond Engl.* (2006) 368:1681–8. 10.1016/S0140-6736(06)69703-1 17098087

[15] Lao Statistics Bureau. *Lao Social Indicator Survey II 2017, Survey Findings Report.* Vientiane: Lao Statistics Bureau and UNICEF (2018).

[16] Harder T Rodekamp E Schellong K Dudenhausen J Plagemann A. Birth weight and subsequent risk of type 2 diabetes: a meta-analysis. *Am J Epidemiol.* (2007) 165:849–57. 10.1093/aje/kwk071 17215379

[17] Hanson M Godfrey K Lillycrop K Burdge G Gluckman P. Developmental plasticity and developmental origins of non-communicable disease: theoretical considerations and epigenetic mechanisms. *Prog Biophys Mol Biol.* (2011) 106:272–80. 10.1016/j.pbiomolbio.2010.12.008 21219925

[18] Lillycrop K Phillips E Torrens C Hanson M Jackson A Burdge G. Feeding pregnant rats a protein-restricted diet persistently alters the methylation of specific cytosines in the hepatic PPAR alpha promoter of the offspring. *Br J Nutr.* (2008) 100:278–82. 10.1017/S0007114507894438 18186951 PMC2564112

[19] Lillycrop K. Effect of maternal diet on the epigenome: implications for human metabolic disease. *Proc Nutr Soc.* (2011) 70:64–72. 10.1017/S0029665110004027 21266093

[20] Indrio F Martini S Francavilla R Corvaglia L Cristofori F Mastrolia S Epigenetic Matters: The Link between Early Nutrition, Microbiome, and Long-term Health Development. *Front Pediatr.* (2017) 5:178. 10.3389/fped.2017.00178 28879172 PMC5572264

[21] Carson C Lawson H. Epigenetics of metabolic syndrome. *Physiol Genomics.* (2018) 50:947–55. 10.1152/physiolgenomics.00072.2018 30240346 PMC6293117

[22] Barker D Winter P Osmond C Margetts B Simmonds S. Weight in infancy and death from ischaemic heart disease. *Lancet Lond Engl.* (1989) 2:577–80. 10.1016/S0140-6736(89)90710-1 2570282

[23] Barker D Osmond C. Infant mortality, childhood nutrition, and ischaemic heart disease in England and Wales. *Lancet Lond Engl.* (1986) 1:1077–81. 10.1016/S0140-6736(86)91340-1 2871345

[24] Hales C Barker D. The thrifty phenotype hypothesis. *Br Med Bull.* (2001) 60:5–20. 10.1093/bmb/60.1.5 11809615

[25] de Boo H Harding J. The developmental origins of adult disease (Barker) hypothesis. *Aust N Z J Obstet Gynaecol.* (2006) 46:4–14. 10.1111/j.1479-828X.2006.00506.x 16441686

[26] Popkin B Corvalan C Grummer-Strawn L. Dynamics of the double burden of malnutrition and the changing nutrition reality. *Lancet Lond Engl.* (2020) 395:65–74. 10.1016/S0140-6736(19)32497-3 31852602 PMC7179702

[27] Prentice A Ward K Goldberg G Jarjou L Moore S Fulford A Critical windows for nutritional interventions against stunting. *Am J Clin Nutr.* (2013) 97:911–8. 10.3945/ajcn.112.052332 23553163 PMC3628381

[28] Castanys-Muñoz E Martin M Vazquez E. Building a Beneficial Microbiome from Birth. *Adv Nutr Bethesda Md.* (2016) 7:323–30. 10.3945/an.115.010694 26980815 PMC4785476

[29] Bäckhed F Ley R Sonnenburg J Peterson D Gordon J. Host-bacterial mutualism in the human intestine. *Science.* (2005) 307:1915–20. 10.1126/science.1104816 15790844

[30] Clemente J Ursell L Parfrey L Knight R. The Impact of the Gut Microbiota on Human Health: An Integrative View. *Cell.* (2012) 148:1258–70. 10.1016/j.cell.2012.01.035 22424233 PMC5050011

[31] Stiemsma L Michels K. The Role of the Microbiome in the Developmental Origins of Health and Disease. *Pediatrics.* (2018) 141:e20172437. 10.1542/peds.2017-2437 29519955 PMC5869344

[32] Arrieta M Stiemsma L Dimitriu P Thorson L Russell S Yurist-Doutsch S Early infancy microbial and metabolic alterations affect risk of childhood asthma. *Sci Transl Med.* (2015) 7:307ra152. 10.1126/scitranslmed.aab2271 26424567

[33] Ridaura V Faith J Rey F Cheng J Duncan A Kau A Gut microbiota from twins discordant for obesity modulate metabolism in mice. *Science.* (2013) 341:1241214. 10.1126/science.1241214 24009397 PMC3829625

[34] Blanton L Charbonneau M Salih T Barratt M Venkatesh S Ilkaveya O Gut bacteria that prevent growth impairments transmitted by microbiota from malnourished children. *Science.* (2016) 351:aad3311. 10.1126/science.aad3311 26912898 PMC4787260

[35] Smith M Yatsunenko T Manary M Trehan I Mkakosya R Cheng J Gut Microbiomes of Malawian Twin Pairs Discordant for Kwashiorkor. *Science.* (2013) 339:548–54. 10.1126/science.1229000 23363771 PMC3667500

[36] Vrieze A Van Nood E Holleman F Salojärvi J Kootte R Bartelsman J Transfer of intestinal microbiota from lean donors increases insulin sensitivity in individuals with metabolic syndrome. *Gastroenterology.* (2012) 143:913–6.e7. 10.1053/j.gastro.2012.06.031 22728514

[37] Tilg H Zmora N Adolph T Elinav E. The intestinal microbiota fuelling metabolic inflammation. *Nat Rev Immunol.* (2020) 20:40–54. 10.1038/s41577-019-0198-4 31388093

[38] Allin K Tremaroli V Caesar R Jensen B Damgaard M Bahl M Aberrant intestinal microbiota in individuals with prediabetes. *Diabetologia.* (2018) 61:810–20. 10.1007/s00125-018-4550-1 29379988 PMC6448993

[39] Petersen C Bell R Klag K Lee S Soto R Ghazaryan A T cell-mediated regulation of the microbiota protects against obesity. *Science.* (2019) 365:eaat9351. 10.1126/science.aat9351 31346040 PMC7294966

[40] Li R Andreu-Sánchez S Kuipers F Fu J. Gut microbiome and bile acids in obesity-related diseases. *Best Pract Res Clin Endocrinol Metab.* (2021) 35:101493. 10.1016/j.beem.2021.101493 33707081

[41] Zhao X Setchell K Huang R Mallawaarachchi I Ehsan L Dobrzykowski Iii E Bile Acid Profiling Reveals Distinct Signatures in Undernourished Children with Environmental Enteric Dysfunction. *J Nutr.* (2021) 151:3689–700. 10.1093/jn/nxab321 34718665 PMC8643614

[42] Vonaesch P Morien E Andrianonimiadana L Sanke H Mbecko J Huus K Stunted childhood growth is associated with decompartmentalization of the gastrointestinal tract and overgrowth of oropharyngeal taxa. *Proc Natl Acad Sci U.S.A.* (2018) 115:E8489–98. 10.1073/pnas.1806573115 30126990 PMC6130352

[43] Ley R Turnbaugh P Klein S Gordon J. Human gut microbes associated with obesity. *Nature.* (2006) 444:1022–3. 10.1038/4441022a 17183309

[44] van Deuren T Blaak E Canfora E. Butyrate to combat obesity and obesity-associated metabolic disorders: Current status and future implications for therapeutic use. *Obes Rev.* (2022) 23:e13498. 10.1111/obr.13498 35856338 PMC9541926

[45] Bridgeman S Northrop W Melton P Ellison G Newsholme P Mamotte C. Butyrate generated by gut microbiota and its therapeutic role in metabolic syndrome. *Pharmacol Res.* (2020) 160:105174. 10.1016/j.phrs.2020.105174 32860943

[46] Coppola S Avagliano C Calignano A Berni Canani R. The Protective Role of Butyrate against Obesity and Obesity-Related Diseases. *Molecules.* (2021) 26:682. 10.3390/molecules26030682 33525625 PMC7865491

[47] Natividad J Lamas B Pham H Michel M Rainteau D Bridonneau C Bilophila wadsworthia aggravates high fat diet induced metabolic dysfunctions in mice. *Nat Commun.* (2018) 9:2802. 10.1038/s41467-018-05249-7 30022049 PMC6052103

[48] Forslund K Hildebrand F Nielsen T Falony G Le Chatelier E Sunagawa S Disentangling type 2 diabetes and metformin treatment signatures in the human gut microbiota. *Nature.* (2015) 528:262–6. 10.1038/nature15766 26633628 PMC4681099

[49] Qin N Yang F Li A Prifti E Chen Y Shao L Alterations of the human gut microbiome in liver cirrhosis. *Nature.* (2014) 513:59–64. 10.1038/nature13568 25079328

[50] Sato K Takahashi N Kato T Matsuda Y Yokoji M Yamada M Aggravation of collagen-induced arthritis by orally administered Porphyromonas gingivalis through modulation of the gut microbiota and gut immune system. *Sci Rep.* (2017) 7:6955. 10.1038/s41598-017-07196-7 28761156 PMC5537233

[51] Lloyd-Price J Arze C Ananthakrishnan A Schirmer M Avila-Pacheco J Poon T Multi-omics of the gut microbial ecosystem in inflammatory bowel diseases. *Nature.* (2019) 569:655–62. 10.1038/s41586-019-1237-9 31142855 PMC6650278

[52] Atarashi K Suda W Luo C Kawaguchi T Motoo I Narushima S Ectopic colonization of oral bacteria in the intestine drives TH1 cell induction and inflammation. *Science.* (2017) 358:359–65. 10.1126/science.aan4526 29051379 PMC5682622

[53] Bauer K Littlejohn P Ayala V Creus-Cuadros A Finlay B. Nonalcoholic Fatty Liver Disease and the Gut-Liver Axis: Exploring an Undernutrition Perspective. *Gastroenterology.* (2022) 162:1858–75.e2. 10.1053/j.gastro.2022.01.058 35248539

[54] Ververs M Antierens A Sackl A Staderini N Captier V. Which Anthropometric Indicators Identify a Pregnant Woman as Acutely Malnourished and Predict Adverse Birth Outcomes in the Humanitarian Context? *PLoS Curr.* (2013) 5:ecurrents.dis.54a8b618c1bc031ea140e3f2934599c8. 10.1371/currents.dis.54a8b618c1bc031ea140e3f2934599c8 23787989 PMC3682760

[55] Hartung C Lerer A Anokwa Y Tseng C Brunette W Borriello G. Open data kit: tools to build information services for developing regions. *Proceedings of the 4th ACM/IEEE International Conference on Information and Communication Technologies and Development – ICTD ’10.* London (2010). 10.1145/2369220.2369236

[56] Callahan B McMurdie P Rosen M Han A Johnson A Holmes S. DADA2: High-resolution sample inference from Illumina amplicon data. *Nat Methods.* (2016) 13:581–3. 10.1038/nmeth.3869 27214047 PMC4927377

[57] Quast C Pruesse E Yilmaz P Gerken J Schweer T Yarza P The SILVA ribosomal RNA gene database project: improved data processing and web-based tools. *Nucleic Acids Res.* (2013) 41:D590–6. 10.1093/nar/gks1219 23193283 PMC3531112

[58] Beghini F McIver L Blanco-Míguez A Dubois L Asnicar F Maharjan S Integrating taxonomic, functional, and strain-level profiling of diverse microbial communities with bioBakery 3. Turnbaugh P, Franco E, Brown CT, editors. *eLife.* (2021) 10:e65088. 10.7554/eLife.65088 33944776 PMC8096432

[59] Segata N Haake S Mannon P Lemon K Waldron L Gevers D Composition of the adult digestive tract bacterial microbiome based on seven mouth surfaces, tonsils, throat and stool samples. *Genome Biol.* (2012) 13:R42. 10.1186/gb-2012-13-6-r42 22698087 PMC3446314

[60] Truong D Franzosa E Tickle T Scholz M Weingart G Pasolli E MetaPhlAn2 for enhanced metagenomic taxonomic profiling. *Nat Methods.* (2015) 12:902–3. 10.1038/nmeth.3589 26418763

[61] Scholz M Ward D Pasolli E Tolio T Zolfo M Asnicar F Strain-level microbial epidemiology and population genomics from shotgun metagenomics. *Nat Methods.* (2016) 13(5):435–8. 10.1038/nmeth.3802 26999001

[62] Jeong A Imboden M Ghantous A Novoloaca A Carsin A Kogevinas M DNA Methylation in Inflammatory Pathways Modifies the Association between BMI and Adult-Onset Non-Atopic Asthma. *Int J Environ Res Public Health.* (2019) 16:E600. 10.3390/ijerph16040600 30791383 PMC6406386

[63] Imboden M Wielscher M Rezwan F Amaral A Schaffner E Jeong A Epigenome-wide association study of lung function level and its change. *Eur Respir J.* (2019) 54:1900457. 10.1183/13993003.00457-2019 31073081 PMC6610463

[64] Aryee M Jaffe A Corrada-Bravo H Ladd-Acosta C Feinberg A Hansen K Minfi: a flexible and comprehensive Bioconductor package for the analysis of Infinium DNA methylation microarrays. *Bioinforma Oxf Engl.* (2014) 30:1363–9. 10.1093/bioinformatics/btu049 24478339 PMC4016708

[65] Triche T Weisenberger D Van Den Berg D Laird P Siegmund K. Low-level processing of Illumina Infinium DNA Methylation BeadArrays. *Nucleic Acids Res.* (2013) 41:e90. 10.1093/nar/gkt090 23476028 PMC3627582

[66] Brägelmann J Lorenzo Bermejo J. A comparative analysis of cell-type adjustment methods for epigenome-wide association studies based on simulated and real data sets. *Brief Bioinform.* (2019) 20:2055–65. 10.1093/bib/bby068 30099476 PMC6954449

[67] Kaushal A Zhang H Karmaus W Ray M Torres M Smith A Comparison of different cell type correction methods for genome-scale epigenetics studies. *BMC Bioinformatics.* (2017) 18:216. 10.1186/s12859-017-1611-2 28410574 PMC5391562

[68] Rahmani E Schweiger R Rhead B Criswell L Barcellos L Eskin E Cell-type-specific resolution epigenetics without the need for cell sorting or single-cell biology. *Nat Commun.* (2019) 10:3417. 10.1038/s41467-019-11052-9 31366909 PMC6668473

[69] Teschendorff A Zheng S. Cell-type deconvolution in epigenome-wide association studies: a review and recommendations. *Epigenomics.* (2017) 9:757–68. 10.2217/epi-2016-0153 28517979

[70] Vujkovic-Cvijin I Sklar J Jiang L Natarajan L Knight R Belkaid Y. Host variables confound gut microbiota studies of human disease. *Nature.* (2020) 587:448–54. 10.1038/s41586-020-2881-9 33149306 PMC7677204

[71] Ferretti P Pasolli E Tett A Asnicar F Gorfer V Fedi S Mother-to-Infant Microbial Transmission from Different Body Sites Shapes the Developing Infant Gut Microbiome. *Cell Host Microbe.* (2018) 24:133–45.e5. 10.1016/j.chom.2018.06.005 30001516 PMC6716579

[72] Yassour M Jason E Hogstrom L Arthur T Tripathi S Siljander H Strain-Level Analysis of Mother-to-Child Bacterial Transmission during the First Few Months of Life. *Cell Host Microbe.* (2018) 24:146–54.e4. 10.1016/j.chom.2018.06.007 30001517 PMC6091882

[73] Wang S Zeng S Egan M Cherry P Strain C Morais E Metagenomic analysis of mother-infant gut microbiome reveals global distinct and shared microbial signatures. *Gut Microbes.* (2021) 13:1911571. 10.1080/19490976.2021.1911571 33960282 PMC8115609

[74] Friend A Craig L Turner S. The prevalence of metabolic syndrome in children: a systematic review of the literature. *Metab Syndr Relat Disord.* (2013) 11:71–80. 10.1089/met.2012.0122 23249214

[75] Yatsunenko T Rey F Manary M Trehan I Dominguez-Bello M Contreras M Human gut microbiome viewed across age and geography. *Nature.* (2012) 486:222–7. 10.1038/nature11053 22699611 PMC3376388

[76] Mancabelli L Milani C Lugli G Turroni F Ferrario C van Sinderen D Meta-analysis of the human gut microbiome from urbanized and pre-agricultural populations. *Environ Microbiol.* (2017) 19:1379–90. 10.1111/1462-2920.13692 28198087

[77] Lin A Bik E Costello E Dethlefsen L Haque R Relman D Distinct distal gut microbiome diversity and composition in healthy children from Bangladesh and the United States. *PloS One.* (2013) 8:e53838. 10.1371/journal.pone.0053838 23349750 PMC3551965

[78] Olm M Dahan D Carter M Merrill B Yu F Jain S Robust variation in infant gut microbiome assembly across a spectrum of lifestyles. *Science.* (2022) 376:1220–3. 10.1126/science.abj2972 35679413 PMC9894631

[79] Sonnenburg Sonnenburg J. The ancestral and industrialized gut microbiota and implications for human health. *Nat Rev Microbiol.* (2019) 17:383–90. 10.1038/s41579-019-0191-8 31089293

[80] Brewster R Tamburini F Asiimwe E Oduaran O Hazelhurst S Bhatt A. Surveying Gut Microbiome Research in Africans: Toward Improved Diversity and Representation. *Trends Microbiol.* (2019) 27:824–35. 10.1016/j.tim.2019.05.006 31178123 PMC6764420

[81] Porras A Brito I. The internationalization of human microbiome research. *Curr Opin Microbiol.* (2019) 50:50–5. 10.1016/j.mib.2019.09.012 31683111 PMC6907006

[82] Allali I Abotsi R Tow L Thabane L Zar H Mulder N Human microbiota research in Africa: a systematic review reveals gaps and priorities for future research. *Microbiome.* (2021) 9:241. 10.1186/s40168-021-01195-7 34911583 PMC8672519

[83] Abdill R Adamowicz E Blekhman R. Public human microbiome data are dominated by highly developed countries. *PLoS Biol.* (2022) 20:e3001536. 10.1371/journal.pbio.3001536 35167588 PMC8846514

[84] Wells J Sawaya A Wibaek R Mwangome M Poullas M Yajnik C The double burden of malnutrition: aetiological pathways and consequences for health. *Lancet Lond Engl.* (2020) 395:75–88. 10.1016/S0140-6736(19)32472-9 31852605 PMC7613491

[85] Hawkes C Ruel M Salm L Sinclair B Branca F. Double-duty actions: seizing programme and policy opportunities to address malnutrition in all its forms. *Lancet Lond Engl.* (2020) 395:142–55. 10.1016/S0140-6736(19)32506-1 31852603

[86] Ford N Behrman J Hoddinott J Maluccio J Martorell R Ramirez-Zea M Exposure to improved nutrition from conception to age 2 years and adult cardiometabolic disease risk: a modelling study. *Lancet Glob Health.* (2018) 6:e875–84. 10.1016/S2214-109X(18)30231-6 30012268 PMC6138451

[87] Ding T Schloss P. Dynamics and associations of microbial community types across the human body. *Nature.* (2014) 509:357–60. 10.1038/nature13178 24739969 PMC4139711

[88] Chen R Kung V Das S Hossain M Hibberd M Guruge J Duodenal Microbiota in Stunted Undernourished Children with Enteropathy. *N Engl J Med.* (2020) 383:321–33. 10.1056/NEJMoa1916004 32706533 PMC7289524

[89] Subramanian S Huq S Yatsunenko T Haque R Mahfuz M Alam M Persistent gut microbiota immaturity in malnourished Bangladeshi children. *Nature.* (2014) 510:417–21. 10.1038/nature13421 24896187 PMC4189846

[90] Gough E Edens T Geum H Baharmand I Gill S Robertson R Maternal fecal microbiome predicts gestational age, birth weight and neonatal growth in rural Zimbabwe. *EBioMedicine.* (2021) 68:103421. 10.1016/j.ebiom.2021.103421 34139432 PMC8217692

