# Supplementary material for: Dynamics and consequences of nutrition-related microbial dysbiosis in early life: study protocol of the VITERBI GUT project
Source: Front Nutr. 2023 May 18;10:1111478. doi: 10.3389/fnut.2023.1111478 (PMC10232750; doi:10.3389/fnut.2023.1111478)
Supplement: Supplementary file 1 [file Table_1.docx]

Supplementary Table 1. References supporting figure 1.

Undernutrition

| Sanitation ↓ | Patlan-Hernandez, 2021(90) |
| --- | --- |
| Socio-economic structures ↓ | Patlan-Hernandez, 2021(90) |
| Protein ↓ | Million, 2016(91) |
| Fat ↓ | Uauy, 2000(92) / reviewed in Michaelsen, 2009(93) |
| Carbohydrate-rich diets ↑ | reviewed in Michaelsen, 2009(93) |
| Antioxidants ↓ | Fechner, 2001(94) |
| Diversity in microbiota ↓ | Patterson, 2022(95) / Nieuwdorp, 2016(96) |
| Microbiota maturity ↓ | Subramanian, 2014(88) / Iddrisu, 2021(97) |
| LPS ↑ | Patterson, 2022(95) |
| Community compartmentalization stomach / duodenum ↑ | Vonaesch, 2018(41) |
| Presence of enteropathogens ↑ | Vonaesch, 2018(41) / Dinh, 2016(98) / MAL-ED network Investigators, 2017(99) |
| Anaerobic bacteria ↓ | Million, 2016(91) |
| Methanogens ↓ | Million, 2016(91) |
| Butyrate producers ↓ | Vonaesch, 2018(41) |
| Diversity in microbiota ↓ | Iddrisu, 2021(97) |
| 7alpha-dehydroxylating bacteria ↓ | Vonaesch, 2018(41) |
| non-12OH-hydroxylated secondary BAs (UDCA, LCA) ↓ | Li, 2022(100) |
| *Lactobacillus, Streptococcus, Enterobacteriaceae* ↑ | Vonaesch, 2018(41) |
| *Oscillospira, Lachnospira, Ruminococcus, Coprococcus, Bifidobacterium, Akkermansia, Christensenellaceae* ↓ | Reviewed in Tilg, 2020(36) / Vonaesch, 2022(101) |
| Infections ↑ | Schaible, 2007(102) / Vonaesch, 2018(41) |
| Fat oxidation ↓ | Frisancho, 2003(103) |
| Fat accumulation ↑ | Prendergast, 2014(104) / Nieuwdorp, 2016(96) |
| Secondary bile acid in duodenum ↓ | Zhao, 2021(40) |
| Secondary bile acids in faeces ↓ | Zhao, 2021(40) |
| Amount and pool of bile acids ≠ | Zhao, 2021(40) |
| Low grade inflammation ↑ | Vonaesch, 2022(101) / Chen, 2020(87) |
| Intestinal permeability ↑ | Vonaesch, 2018(41) |
| Oxidative stress ↑ | Preidis, 2014(105) |
| Risk of MetS ↑ | Grey, 2021(106) |
| Risk of hepatic steatosis ↑ | Bauer, 2020(107) |
| Insulin resistance ↑ | Francis, 2017(108) / Jacovetti, 2022(109) |
| Hypertension ↑ | Sawaya, 2005(110) |
| Dyslipidemia ↑ | Prendergast, 2014(104) |
| Mental development ↓ | Prendergast, 2014(104) |
| Pancreatic B cells function ↓ | Jacovetti, 2022(109) |
| Malabsorption ↑ | Million, 2017(111) |
| DNA methylation and expression levels of key metabolic genes ≠ | Ramos-Lopez, 2019(112) |
| Prevalence of anemia ↑ | Veiga, 2010(113) |
| Vitamin deficiency ↑ | Million, 2016(91) |
| Ferritin ↑ | Coyne, 2006(114) |
| White blood cell number ↓ | St clair, 2020(115) |
| BCAA serum level ↓ | Vonaesch, 2022(101) / Semba, 2016(116) |
| Triglyceride serum level ↓↑ | Ogunkeye, 1992(117) / Veiga, 2010(113) |

Overnutrition

| Sanitation ↓ | Leocadio, 2021(118) |
| --- | --- |
| Socio-economic structures ↓ | Mathur, 2019(119) / Leocadio, 2021(118) |
| Protein ↑ | Leocadio, 2021(118) |
| Fat ↑ | Leocadio, 2021(118) |
| Animal-based fatty acids ↑ | Moussavi, 2008(120) |
| Carbohydrate-rich diets ↑ | Leocadio, 2021(118) |
| Fibers ↓ | Seljak, 2021(121) |
| Diversity in microbiota ↓ | Patterson, 2022(95) |
| LPS ↑ | Pendyala, 2012(122) |
| Bacteroides ↓ | Reviewed in He, 2021(123) |
| Presence of enteropathogens ↑ | Wotzka, 2019(124) |
| Ratio Firmicutes/Bacteroidetes ↑ | Magne, 2020(125) |
| Anaerobic bacteria ↓ | Million, 2012(126) |
| Methanogens ↓ | Million, 2012(126) |
| Butyrate producers ↓ | Coppola, 2021(45) |
| Levels of total short chain fatty acids ↑ | Schwiertz, 2010(127) |
| *Lactobacillus, Streptococcus, Enterobacteriaceae* ↑ | Tilg, 2020(36) |
| *Blautia* ↑ | Tilg, 2020(36) |
| *Oscillospira, Lachnospira, Ruminococcus, Coprococcus, Bifidobacterium, Akkermansia, Christensenellaceae* ↓ | Tilg, 2020(36) |
| Infections ↑ | Schaible, 2007(102) |
| Fat accumulation ↑ | Singla, 2010(128) |
| Secondary bile acids in faeces ↓↑ | Wan, 2010(129) |
| Amount and pool of bile acids ≠ | Liaset, 2011(130) |
| Low grade inflammation ↑ | Calder, 2011(131) / Cani, 2007(2) |
| Intestinal permeability ↑ | He, 2021(123) |
| Oxidative stress ↑ | Mastorci, 2017(132) |
| Insulin resistance ↑ | Calder, 2011(131) / Cani, 2007(2) |
| Hypertension ↑ | Berchtold, 1938(133) / Jiang, 2016(134) |
| Dyslipidemia ↑ | Grundy, 2016(135) |
| Prevalence of anemia ↑ | Alshwaiyat, 2021(136) |
| Vitamin deficiency ↑ | Vranic, 2019(137) |
| Ferritin ↑ | Khan, 2016(138) |
| BCAA serum level ↑ | She, 2007(139) |
| Triglyceride serum level ↑ | Kenneth, 2020(140) |
| Free fatty acid serum level ↑ | Kenneth, 2020(140) |

91. Million M, Tidjani Alou M, Khelaifia S, Bachar D, Lagier JC, Dione N, et al. Increased Gut Redox and Depletion of Anaerobic and Methanogenic Prokaryotes in Severe Acute Malnutrition. Sci Rep. 2016 May 17;6(1):26051.

92. Uauy R, Mize CE, Castillo-Duran C. Fat intake during childhood: metabolic responses and effects on growth. Am J Clin Nutr. 2000 Nov;72(5 Suppl):1354S-1360S.

93. Michaelsen KF, Hoppe C, Roos N, Kaestel P, Stougaard M, Lauritzen L, et al. Choice of foods and ingredients for moderately malnourished children 6 months to 5 years of age. Food Nutr Bull. 2009 Sep;30(3 Suppl):S343-404.

94. Fechner A, Böhme CC, Gromer S, Funk M, Schirmer RH, Becker K. Antioxidant Status and Nitric Oxide in the Malnutrition Syndrome Kwashiorkor. Pediatr Res. 2001 Feb;49(2):237–43.

95. Patterson GT, Osorio EY, Peniche A, Dann SM, Cordova E, Preidis GA, et al. Pathologic Inflammation in Malnutrition Is Driven by Proinflammatory Intestinal Microbiota, Large Intestine Barrier Dysfunction, and Translocation of Bacterial Lipopolysaccharide. Front Immunol [Internet]. 2022 [cited 2022 Aug 18];13. Available from: https://www.frontiersin.org/articles/10.3389/fimmu.2022.846155

96. de Clercq NC, Groen AK, Romijn JA, Nieuwdorp M. Gut Microbiota in Obesity and Undernutrition. Adv Nutr Bethesda Md. 2016 Nov;7(6):1080–9.

97. Iddrisu I, Monteagudo-Mera A, Poveda C, Pyle S, Shahzad M, Andrews S, et al. Malnutrition and Gut Microbiota in Children. Nutrients. 2021 Aug 8;13(8):2727.

98. Dinh DM, Ramadass B, Kattula D, Sarkar R, Braunstein P, Tai A, et al. Longitudinal Analysis of the Intestinal Microbiota in Persistently Stunted Young Children in South India. PloS One. 2016;11(5):e0155405.

99. MAL-ED Network Investigators. Relationship between growth and illness, enteropathogens and dietary intakes in the first 2 years of life: findings from the MAL-ED birth cohort study. BMJ Glob Health. 2017;2(4):e000370.

100. Li M, Wang S, Li Y, Zhao M, Kuang J, Liang D, et al. Gut microbiota-bile acid crosstalk contributes to the rebound weight gain after calorie restriction in mice. Nat Commun. 2022 Dec;13(1):2060.

101. Vonaesch P, Araújo JR, Gody JC, Mbecko JR, Sanke H, Andrianonimiadana L, et al. Stunted children display ectopic small intestinal colonization by oral bacteria, which cause lipid malabsorption in experimental models. Proc Natl Acad Sci. 2022 Oct 11;119(41):e2209589119.

102. Schaible UE, Kaufmann SHE. Malnutrition and Infection: Complex Mechanisms and Global Impacts. PLoS Med. 2007 May;4(5):e115.

103. Frisancho AR. Reduced rate of fat oxidation: A metabolic pathway to obesity in the developing nations. Am J Hum Biol. 2003;15(4):522–32.

104. Prendergast AJ, Humphrey JH. The stunting syndrome in developing countries. Paediatr Int Child Health. 2014 Apr;34(4):250–65.

105. Preidis GA, Keaton MA, Campeau PM, Bessard BC, Conner ME, Hotez PJ. The Undernourished Neonatal Mouse Metabolome Reveals Evidence of Liver and Biliary Dysfunction, Inflammation, and Oxidative Stress. J Nutr. 2014 Mar 1;144(3):273–81.

106. Grey K, Gonzales GB, Abera M, Lelijveld N, Thompson D, Berhane M, et al. Severe malnutrition or famine exposure in childhood and cardiometabolic non-communicable disease later in life: a systematic review. BMJ Glob Health. 2021 Mar 1;6(3):e003161.

107. Bauer KC, Huus KE, Brown EM, Bozorgmehr T, Petersen C, Cirstea MS, et al. Dietary Intervention Reverses Fatty Liver and Altered Gut Microbiota during Early-Life Undernutrition. mSystems. 2020 Sep 8;5(5):e00499-20.

108. Francis NK, Pawar HS, Mitra A, Mitra A. Assessment of Insulin Sensitivity and its Convalescence with Dietary Rehabilitation in Undernourished Rural West Bengal Population. J Clin Diagn Res JCDR. 2017 May;11(5):LC29–32.

109. Jacovetti C, Regazzi R. Mechanisms Underlying the Expansion and Functional Maturation of β-Cells in Newborns: Impact of the Nutritional Environment. Int J Mol Sci. 2022 Feb 14;23(4):2096.

110. Sawaya AL, Sesso R, Florêncio TM de MT, Fernandes MTB, Martins PA. Association between chronic undernutrition and hypertension. Matern Child Nutr. 2005 Jul;1(3):155–63.

111. Million M, Diallo A, Raoult D. Gut microbiota and malnutrition. Microb Pathog. 2017 May;106:127–38.

112. Ramos-Lopez O, Riezu-Boj JI, Milagro FI, Martinez JA. Epigenetics of Undernutrition. In: Patel VB, Preedy VR, editors. Handbook of Nutrition, Diet, and Epigenetics [Internet]. Cham: Springer International Publishing; 2019 [cited 2022 Aug 23]. p. 457–81. Available from: https://doi.org/10.1007/978-3-319-55530-0_24

113. Veiga GRS, Ferreira HS, Sawaya AL, Calado J, Florêncio TMMT. Dyslipidaemia and Undernutrition in Children from Impoverished Areas of Maceió, State of Alagoas, Brazil. Int J Environ Res Public Health. 2010 Dec;7(12):4139–51.

114. Coyne D. Iron indices: What do they really mean? Kidney Int. 2006 May 1;69:S4–8.

115. Symptoms [Internet]. St. Clair Health. [cited 2022 Nov 10]. Available from: https://www.stclair.org/services/mayo-clinic-health-information/symptoms/

116. Semba RD, Shardell M, Sakr Ashour FA, Moaddel R, Trehan I, Maleta KM, et al. Child Stunting is Associated with Low Circulating Essential Amino Acids. EBioMedicine. 2016 Apr;6:246–52.

117. Ogunkeye OO, Ighogboja IS. Increase in total serum triglyceride and phospholipid in kwashiorkor. Ann Trop Paediatr. 1992;12(4):463–6.

118. Leocádio PCL, Lopes SC, Dias RP, Alvarez-Leite JI, Guerrant RL, Malva JO, et al. The Transition From Undernutrition to Overnutrition Under Adverse Environments and Poverty: The Risk for Chronic Diseases. Front Nutr. 2021 Apr 23;8:676044.

119. Mathur P, Pillai R. Overnutrition: Current scenario & combat strategies. Indian J Med Res. 2019 Jun;149(6):695–705.

120. Moussavi N, Gavino V, Receveur O. Is obesity related to the type of dietary fatty acids? An ecological study. Public Health Nutr. 2008 Nov;11(11):1149–55.

121. Seljak BK, Valenčič E, Hristov H, Hribar M, Lavriša Ž, Kušar A, et al. Inadequate Intake of Dietary Fibre in Adolescents, Adults, and Elderlies: Results of Slovenian Representative SI. Menu Study. Nutrients. 2021 Nov;13(11):3826.

122. S P, Jm W, Pr H. A high-fat diet is associated with endotoxemia that originates from the gut. Gastroenterology [Internet]. 2012 May [cited 2022 Nov 24];142(5). Available from: https://pubmed.ncbi.nlm.nih.gov/22326433/

123. He L. Alterations of Gut Microbiota by Overnutrition Impact Gluconeogenic Gene Expression and Insulin Signaling. Int J Mol Sci. 2021 Feb 20;22(4):2121.

124. Wotzka SY, Kreuzer M, Maier L, Arnoldini M, Nguyen BD, Brachmann AO, et al. Escherichia coli limits Salmonella Typhimurium infections after diet shifts and fat-mediated microbiota perturbation in mice. Nat Microbiol. 2019 Dec;4(12):2164–74.

125. Magne F, Gotteland M, Gauthier L, Zazueta A, Pesoa S, Navarrete P, et al. The Firmicutes/Bacteroidetes Ratio: A Relevant Marker of Gut Dysbiosis in Obese Patients? Nutrients. 2020 May;12(5):1474.

126. Million M, Maraninchi M, Henry M, Armougom F, Richet H, Carrieri P, et al. Obesity-associated gut microbiota is enriched in Lactobacillus reuteri and depleted in Bifidobacterium animalis and Methanobrevibacter smithii. Int J Obes. 2012 Jun;36(6):817–25.

127. Schwiertz A, Taras D, Schäfer K, Beijer S, Bos NA, Donus C, et al. Microbiota and SCFA in lean and overweight healthy subjects. Obes Silver Spring Md. 2010 Jan;18(1):190–5.

128. Singla P, Bardoloi A, Parkash AA. Metabolic effects of obesity: A review. World J Diabetes. 2010 Jul 15;1(3):76–88.

129. Wan Y, Yuan J, Li J, Li H, Zhang J, Tang J, et al. Unconjugated and secondary bile acid profiles in response to higher-fat, lower-carbohydrate diet and associated with related gut microbiota: A 6-month randomized controlled-feeding trial. Clin Nutr. 2020 Feb 1;39(2):395–404.

130. Liaset B, Hao Q, Jørgensen H, Hallenborg P, Du ZY, Ma T, et al. Nutritional regulation of bile acid metabolism is associated with improved pathological characteristics of the metabolic syndrome. J Biol Chem. 2011 Aug 12;286(32):28382–95.

131. Calder PC, Ahluwalia N, Brouns F, Buetler T, Clement K, Cunningham K, et al. Dietary factors and low-grade inflammation in relation to overweight and obesity. Br J Nutr. 2011 Dec;106 Suppl 3:S5-78.

132. Mastorci F, Vassalle C, Chatzianagnostou K, Marabotti C, Siddiqui K, Eba AO, et al. Undernutrition and Overnutrition Burden for Diseases in Developing Countries: The Role of Oxidative Stress Biomarkers to Assess Disease Risk and Interventional Strategies. Antioxidants. 2017 Jun 8;6(2):41.

133. Berchtold P, Sims EA, Horton ES, Berger M. Obesity and hypertension: epidemiology, mechanisms, treatment. Biomed Pharmacother Biomedecine Pharmacother. 1983;37(6):251–8.

134. Jiang SZ, Lu W, Zong XF, Ruan HY, Liu Y. Obesity and hypertension. Exp Ther Med. 2016 Oct;12(4):2395–9.

135. Grundy SM. Overnutrition, ectopic lipid and the metabolic syndrome. J Investig Med. 2016 Aug 1;64(6):1082–6.

136. Alshwaiyat NM, Ahmad A, Wan Hassan WMR, Al-Jamal HAN. Association between obesity and iron deficiency (Review). Exp Ther Med. 2021 Nov;22(5):1268.

137. Vranić L, Mikolašević I, Milić S. Vitamin D Deficiency: Consequence or Cause of Obesity? Medicina (Mex). 2019 Aug 28;55(9):541.

138. Khan A, Khan WM, Ayub M, Humayun M, Haroon M. Ferritin Is a Marker of Inflammation rather than Iron Deficiency in Overweight and Obese People. J Obes. 2016;2016:1937320.

139. She P, Van Horn C, Reid T, Hutson SM, Cooney RN, Lynch CJ. Obesity-related elevations in plasma leucine are associated with alterations in enzymes involved in branched-chain amino acid metabolism. Am J Physiol Endocrinol Metab. 2007 Dec;293(6):E1552-1563.

140. Feingold KR. Obesity and Dyslipidemia. In: Feingold KR, Anawalt B, Boyce A, Chrousos G, de Herder WW, Dhatariya K, et al., editors. Endotext [Internet]. South Dartmouth (MA): MDText.com, Inc.; 2000 [cited 2022 Nov 10]. Available from: http://www.ncbi.nlm.nih.gov/books/NBK305895/
